# Supplementary material for: LensAge index as a deep learning-based biological age for self-monitoring the risks of age-related diseases and mortality
Source: Nat Commun. 2023 Nov 6;14:7126. doi: 10.1038/s41467-023-42934-8 (PMC10628111; doi:10.1038/s41467-023-42934-8)
Supplement: Supplementary file 1 — Supplementary Information [file 41467_2023_42934_MOESM1_ESM.pdf]

Supplementary Information
Supplementary Figures

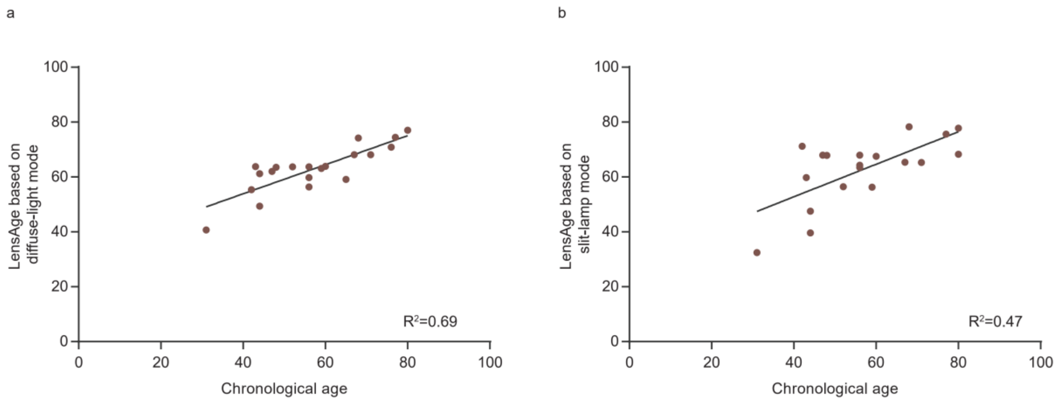

Supplementary Figure 1. The LensAge estimation among the non-Chinese population. a, Scatterplot shows the correlation of LensAge at the individual level with chronological age for relatively healthy participants for diffuse-light mode (*p*=5.28e-6, two-sided linear regression, n=20). **b,** Scatterplot shows the correlation of LensAge at the individual level with chronological age for relatively healthy participants for slit-lamp mode (*p*=1.17e-3, two-sided linear regression, n=19).

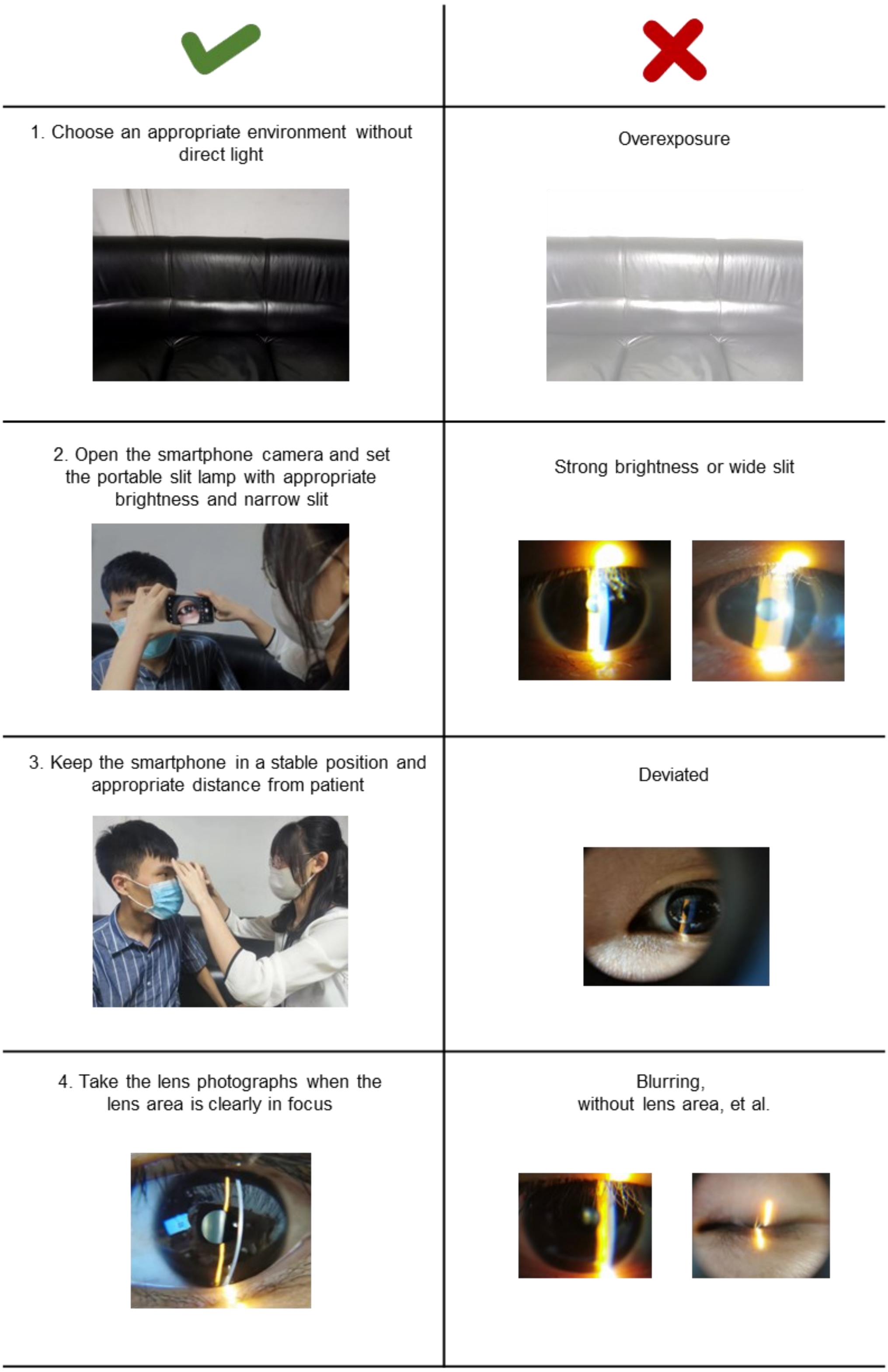

Supplementary Figure 2. Brief instructions for capturing lens photographs using smartphones.

Supplementary Tables

Supplementary Table 1 | The performance of four trained CNNs in the reference dataset consisting of traditional slit lamp images

	Image level	Individual level		
	**MAE**	**MAE**	R^2	***p* value**
**Diffuse-light mode**				
InceptionV3	4.88	4.25	0.89	<1.00e-36*
ResNet50	5.10	4.39	0.88	<1.00e-36*
DenseNet	4.89	4.22	0.89	<1.00e-36*
InceptionResnetV2	5.54	4.80	0.86	<1.00e-36*
**Slit-lamp mode**				
InceptionV3	5.25	4.82	0.82	<1.00e-36*
ResNet50	6.18	5.45	0.76	<1.00e-36*
DenseNet	5.34	4.83	0.82	<1.00e-36*
InceptionResnetV2	5.25	4.82	0.82	<1.00e-36*

CNN, convolutional neural network; MAE, mean absolute error. *p* values from two-sided tests using linear regressions. **p* value<0.001.

Supplementary Table 2 | LensAge prediction for different types of cataracts

	Cortical cataracts			Nuclear cataracts			Subcapsular cataracts		
**LensAge mode**	**MAE**	**R^2**	***p* value**	**MAE**	**R^2**	***p* value**	**MAE**	**R^2**	***p* value**
Diffuse-light mode	4.66	0.68	<1.00e-36*	4.78	0.64	<1.00e-36*	4.51	0.73	4.68e-21*
Slit-lamp mode	4.54	0.73	<1.00e-36*	5.90	0.58	<1.00e-36*	4.38	0.80	5.01e-29*

MAE, mean absolute error. *p* values from two-sided tests using linear regressions. **p* value<0.05.

Supplementary Table 3 | Performance of different methods for assessing biological age in humans found in the literature

Research	Type of data	Source	Age group (years)	Error (years)	Age-related metric
This study	Lens imaging	Diffuse-light and slit-lamp images	20-96	4.3-4.8	Age-related disease risks and all-cause mortality risk
Hannum *et al*[1]	DNA methylation	Whole blood	19-101	4.9	Age-associated gene expression
Horvath[2]	DNA methylation	Heterogeneous tissues	0-101	3.6	Aging changes in multiple tissues and cell types
Peters *et al*[3]	Transcriptomics	Peripheral blood mononuclear cells	Mean 28.4-72.2	7.8	Blood pressure, cholesterol levels, fasting glucose, and body mass index
Fleischer *et al*[4]	Transcriptomics	Dermal fibroblasts	1-94	7.7	Progeria
Putin *et al*[5]	Blood profiles	Common blood biochemistry and cell count tests	1-100	5.6	Albumin, glucose, alkaline phosphatase, urea, and erythrocytes
Mamoshina *et al*[6]	Blood profiles	Common blood biochemistry and cell count tests	Interquartile range 32-64	5.9	All-cause mortality risk
Sayed *et al*[7]	Blood immunome	Peripheral blood mononuclear cells or whole blood	8-96	15.2	Systemic age-related inflammation
Liem *et al*[8]	Brain imaging	Magnetic resonance imaging (MRI) data	19-82	4.3	Cognitive impairment

Chen *et al*[9]	3D facial morphologies	3D facial images	17-77	6.1-6.2	Albumin, uric acid, total cholesterol, and low-density lipoprotein cholesterol levels
Zhu *et al*[10]	Fundus imaging	Retinal fundus and optical coherence tomography images	40-69	3.6	All-cause mortality risk

Supplementary Table 4 | Analyses of the importance of different lens structures on LensAge prediction for different types of cataracts

Masking structure	MAE			Adjusted OR (95% CI)	*p* value
	Cortical cataract	Nuclear cataract	Subcapsular cataract		
Lens cortex	8.47	5.44	4.10	1.23 (1.16-1.31)	6.72e-11*
Lens nucleus	5.95	11.43	4.86	1.24 (1.16-1.33)	1.55e-10*
Lens capsule	5.61	4.25	5.84	1.12 (1.02-1.22)	1.60e-2*

Adjusted logistic regression models were used to compare the influence of masking the cortex on predictive errors between cortical cataracts and other cataracts, the influence of masking the nucleus on predictive errors between nuclear cataracts and other cataracts, and the influence of masking the capsule on predictive errors between subcapsular cataracts and other cataracts. The results are reported with adjusted ORs. MAE, mean absolute error; OR, odds ratio; CI, confidence interval. p values from two-sided tests using adjusted logistic regressions. **p* value<0.001.

Supplementary Table 5 | The distribution of the LensAge index at the individual level among the general population

	Diffuse-light mode	Slit-lamp mode
**LensAge index (years, mean±s.d.)**	2.0±5.9	2.9±7.1
**Distribution of the LensAge index (years)**		
Minimum	-29.9	-35.8
25th percentile	-2.0	-1.7
Median	1.9	2.5
75th percentile	6.0	7.2
Maximum	27.8	33.4
**Proportions of fast agers**		
≥5 years	30.5%	35.5%
≥10 years	8.3%	14.7%
≥20 years	0.4%	1.3%

Supplementary Table 6 | Subgroup analyses of the comparison of age-related changes between individuals with negative/positive LensAge index for diffuse-light mode.

	<60 years old		≥60 years old	
	**Adjusted odds ratio (95% CI)**	***p* value**	**Adjusted odds ratio (95% CI)**	***p* value**
**Eye aging**				
Moderate or severe visual impairment	3.14 (1.86-5.32)	2.10e-5*	1.76 (1.34-2.31)	5.80e-5*
Senile cataracts	8.91 (5.93-13.37)	4.86e-26*	0.97 (0.76-1.22)	0.769
Vitreous opacity	1.02 (0.44-2.34)	0.970	2.47 (1.54-3.97)	1.87e-4*
**Systemic aging**				
Age-related chronic diseases	3.41 (1.79-6.49)	1.95e-4*	1.16 (0.94-1.43)	0.161
Age-related findings of chest X-ray	0.99 (0.55-1.77)	0.962	1.88 (1.42-2.50)	1.40e-5*
Age-related findings of electrocardiograms	1.51 (0.98-2.31)	0.059	1.30 (1.08-1.57)	0.007*

CI=confidence interval. *p* values from two-sided tests using adjusted logistic regressions. *p value<0.05.

Supplementary Table 7 | Subgroup analyses of the association of the LensAge index with age-related changes in individuals with positive LensAge index for diffuse-light mode

	**<60 years old**		**≥60 years old**	
	**Adjusted odds ratio (95% CI)**	***p* value**	**Adjusted odds ratio (95% CI)**	***p* value**
**Eye aging**				
Moderate or severe visual impairment	1.17 (1.10-1.24)	4.25e-7*	1.13 (1.08-1.19)	3.00e-6*
Senile cataracts	1.20 (1.13-1.27)	1.50e-9*	1.10 (1.05-1.16)	9.30e-5*
Vitreous opacity	0.99 (0.92-1.06)	0.675	1.11 (1.02-1.20)	0.012*
**Systemic aging**				
Age-related chronic diseases	1.17 (1.12-1.22)	3.88e-12*	1.05 (1.01-1.09)	0.010*
Age-related findings of chest X-ray	1.07 (1.01-1.12)	0.018*	1.08 (1.03-1.13)	0.003*
Age-related findings of electrocardiograms	1.03 (1.00-1.07)	0.094	1.02 (0.99-1.06)	0.184

CI=confidence interval. *p* values from two-sided tests using adjusted logistic regressions. **p* value<0.05.

Supplementary Table 8 | Subgroup analyses of the comparison of age-related changes between individuals with negative/positive LensAge index for slit-lamp mode

	<60 years old		≥60 years old	
	**Adjusted odds ratio (95% CI)**	***p* value**	**Adjusted odds ratio (95% CI)**	***p* value**
**Eye aging**				
Moderate or severe visual impairment	2.36 (1.39-4.00)	0.001*	2.22 (1.69-2.90)	6.89e-9*
Senile cataracts	1.84 (1.19-2.86)	0.007*	0.83 (0.65-1.05)	0.122
Vitreous opacity	1.43 (0.60-3.39)	0.420	1.52 (0.99-2.33)	0.590
**Systemic aging**				
Age-related chronic diseases	1.40 (0.84-2.34)	0.201	1.11 (0.91-1.35)	0.323
Age-related findings of chest X-ray	1.10 (0.59-2.08)	0.759	1.19 (0.90-1.58)	0.222
Age-related findings of electrocardiograms	1.44 (0.93-2.21)	0.101	1.09 (0.91-1.31)	0.357

CI=confidence interval. *p* values from two-sided tests using adjusted logistic regressions. **p* value<0.05.

Supplementary Table 9 | Subgroup analyses of the association of the LensAge index with age-related changes in individuals with positive LensAge index for slit-lamp mode

	<60 years old		≥60 years old	
	**Adjusted odds ratio (95% CI)**	***p* value**	**Adjusted odds ratio (95% CI)**	***p* value**
**Eye aging**				
Moderate or severe visual impairment	1.11 (1.07-1.16)	1.00e-6*	1.10 (1.06-1.15)	6.00e-6*
Senile cataracts	1.08 (1.04-1.12)	1.80e-5*	1.01 (0.98-1.05)	0.441
Vitreous opacity	1.00 (0.95-1.05)	0.930	1.06 (1.00-1.12)	0.070
**Systemic aging**				
Age-related chronic diseases	1.05 (1.02-1.08)	0.002*	1.03 (1.00-1.06)	0.066
Age-related findings of chest X-ray	1.04 (1.00-1.09)	0.076	1.09 (1.05-1.13)	5.00e-6*
Age-related findings of electrocardiograms	1.03 (1.00-1.06)	0.064	1.04 (1.01-1.06)	0.010*

CI=confidence interval. *p* values from two-sided tests using adjusted logistic regressions. **p* value<0.05.

Supplementary Table 10 | Comparison of age-related changes between the individuals with LensAge results of <60 and ≥60 years old

	Diffuse-light mode		Slit-lamp mode	
	**Adjusted odds ratio (95% CI)**	***p* value**	**Adjusted odds ratio (95% CI)**	***p* value**
**Eye aging**				
Moderate or severe visual impairment	1.38 (0.98-1.92)	0.062	1.72 (1.24-2.38)	1.20e-3*
Senile cataracts	2.26 (1.71-2.99)	1.03e-8*	1.97 (1.49-2.60)	1.95e-6*
Vitreous opacity	1.07 (0.63-1.81)	0.805	1.19 (0.72-1.96)	0.493
**Systemic aging**				
Age-related chronic diseases	2.00 (1.48-2.69)	5.09e-6*	1.36 (1.02-1.79)	0.034*
The age-related findings of chest X-ray	0.94 (0.66-1.33)	0.723	1.08 (0.76-1.56)	0.662
The age-related findings of electrocardiograms	1.21 (0.94-1.55)	0.143	0.99 (0.78-1.27)	0.955

CI=confidence interval. *p* values from two-sided tests using adjusted logistic regressions. **p* value<0.05.

Supplementary Table 11 | Risks of age-related changes for the LensAge index in the highest quartile versus the LensAge index in the second and third quartiles

	Diffuse-light mode		Slit-lamp mode	
	**Adjusted odds ratio (95% CI)**	***p* value**	**Adjusted odds ratio (95% CI)**	***p* value**
**Eye aging**				
Moderate or severe visual impairment	3.00 (2.28-3.95)	3.67e-15*	2.32 (1.77-3.05)	1.18e-9*
Senile cataracts	2.61 (2.02-3.37)	1.54e-13*	1.35 (1.07-1.71)	1.29e-2*
Vitreous opacity	1.50 (0.98-2.28)	6.04e-2	1.57 (1.04-2.35)	3.07e-2*
**Systemic aging**				
Age-related chronic diseases	1.61 (1.31-1.97)	5.46e-6*	1.23 (1.00-1.53)	4.73e-2*
The age-related findings of chest X-ray	1.50 (1.15-1.94)	2.49e-3*	1.13 (0.87-1.47)	3.63e-1
The age-related findings of electrocardiograms	1.42 (1.18-1.72)	2.69e-4*	1.08 (0.89-1.30)	4.54e-1

CI=confidence interval. *p* values from two-sided tests using adjusted logistic regressions. **p* value<0.05.

Supplementary Table 12 | The association of the LensAge index with blood glucose level

LensAge mode	All ages β (95% CI)	All ages *p* value	<60 years old β (95% CI)	<60 years old *p* value	≥60 years old β (95% CI)	≥60 years old *p* value
Diffuse-light mode	0.04 (0.01-0.07)	0.006*	0.08 (0.03-0.12)	0.001*	0.004 (-0.04-0.05)	0.873
Slit-lamp mode	-0.01 (-0.030-0.018)	0.623	0.02 (-0.02-0.05)	0.352	-0.03 (-0.07-0.01)	0.089

CI=confidence interval. *p* values from two-sided tests using adjusted linear regressions. **p* value<0.05.

Supplementary Table 13 | Analyses of the predictive performance of the LensAge index for all-cause mortality

LensAge index	Adjusted HR	95% CI		*p* value
		Lower	Upper	
Diffuse-light mode	1.08	1.01	1.15	2.43e-2*
Slit-lamp mode	1.07	1.01	1.12	1.21e-2*

HR=hazard rate; CI=confidence interval. *p* values from two-sided tests using adjusted Cox proportional hazards regressions. *p value<0.05.

Supplementary Table 14 | Distribution of images captured by different traditional slit lamps in the datasets

	Reference dataset	Analysis dataset
**Diffuse-light images, n (%)**	4,542 (100%)	5,641 (100%)
BQ-900	1,339 (29.5%)	1,990 (35.3%)
BX-900	1,231 (27.1%)	1,074 (19.0%)
OVS-II	1,013 (22.3%)	1,009 (17.9%)
PSL-Classic	959 (21.1%)	1,568 (27.8%)
**Slit-lamp images, n (%)**	3,713 (100%)	5,663 (100%)
BQ-900	1,089 (29.3%)	1,689 (29.8%)
BX-900	1,101 (29.7%)	1,741 (30.7%)
OVS-II	993 (26.7%)	1,011 (17.9%)
PSL-Classic	530 (14.3%)	1,222 (21.6%)

Supplementary Table 15 | Comparison of baseline characteristics between those who were followed up and those who were lost to follow-up

	Follow-up	Lost to follow-up	*p* value
**No. of participants**	2,982	451	-
**Chronological age in years (mean±s.d.)**	65.9±11.5	66.7±11.3	0.19[a]
**Sex, n (%)**			
Male	1,287	195	0.98[b]
Female	1,695	256	
**LensAge index in years (mean±s.d.)**			
Diffuse-light mode	2.0±5.9	2.3±5.9	0.32[a]
Slit-lamp mode	2.8±7.1	2.9±6.8	0.79[a]

[a]*p* values from two-sided tests using Student's t-tests; [b]*p* value from a two-sided test using χ^2 test.

Supplementary References:

1 Hannum, G. *et al.* Genome-wide methylation profiles reveal quantitative views of human aging rates. *Molecular cell* **49**, 359-367 (2013).

2 Horvath, S. DNA methylation age of human tissues and cell types. *Genome biology* **14**, 1-20 (2013).

3 Peters, M. J. *et al.* The transcriptional landscape of age in human peripheral blood. *Nature communications* **6**, 1-14 (2015).

4 Fleischer, J. G. *et al.* Predicting age from the transcriptome of human dermal fibroblasts. *Genome Biol* **19**, 221, doi:10.1186/s13059-018-1599-6 (2018).

5 Putin, E. *et al.* Deep biomarkers of human aging: application of deep neural networks to biomarker development. *Aging (Albany NY)* **8**, 1021 (2016).

6 Mamoshina, P. *et al.* Population specific biomarkers of human aging: a big data study using South Korean, Canadian, and Eastern European patient populations. *The Journals of Gerontology: Series A* **73**, 1482-1490 (2018).

7 Sayed, N. *et al.* An inflammatory aging clock (iAge) based on deep learning tracks multimorbidity, immunosenescence, frailty and cardiovascular aging. *Nat Aging* **1**, 598-615, doi:10.1038/s43587-021-00082-y (2021).

8 Liem, F. *et al.* Predicting brain-age from multimodal imaging data captures cognitive impairment. *Neuroimage* **148**, 179-188 (2017).

9 Chen, W. *et al.* Three-dimensional human facial morphologies as robust aging markers. *Cell research* **25**, 574-587 (2015).

10 Zhu, Z. *et al.* Retinal age gap as a predictive biomarker for mortality risk. *British Journal of Ophthalmology* (2022).
