# Supplementary material for: LensAge index as a deep learning-based biological age for self-monitoring the risks of age-related diseases and mortality
Source: Nat Commun. 2023 Nov 6;14:7126. doi: 10.1038/s41467-023-42934-8 (PMC10628111; doi:10.1038/s41467-023-42934-8)
Supplement: Supplementary file 3 — Reporting Summary [file 41467_2023_42934_MOESM3_ESM.pdf]

nature portfolio

Corresponding author(s): H.L.

Last updated by author(s): Sep 1, 2023

Reporting Summary

Nature Portfolio wishes to improve the reproducibility of the work that we publish. This form provides structure for consistency and transparency in reporting. For further information on Nature Portfolio policies, see our Editorial Policies and the Editorial Policy Checklist.

Statistics

For all statistical analyses, confirm that the following items are present in the figure legend, table legend, main text, or Methods section.

n/a	Confirmed	
☐	☒	The exact sample size (*n*) for each experimental group/condition, given as a discrete number and unit of measurement
☐	☒	A statement on whether measurements were taken from distinct samples or whether the same sample was measured repeatedly
☐	☒	The statistical test(s) used AND whether they are one- or two-sided<br>*Only common tests should be described solely by name; describe more complex techniques in the Methods section.*
☐	☒	A description of all covariates tested
☐	☒	A description of any assumptions or corrections, such as tests of normality and adjustment for multiple comparisons
☐	☒	A full description of the statistical parameters including central tendency (e.g. means) or other basic estimates (e.g. regression coefficient) AND variation (e.g. standard deviation) or associated estimates of uncertainty (e.g. confidence intervals)
☐	☒	For null hypothesis testing, the test statistic (e.g. *F*, *t*, *r*) with confidence intervals, effect sizes, degrees of freedom and *P* value noted<br>*Give P values as exact values whenever suitable.*
☒	☐	For Bayesian analysis, information on the choice of priors and Markov chain Monte Carlo settings
☐	☒	For hierarchical and complex designs, identification of the appropriate level for tests and full reporting of outcomes
☐	☒	Estimates of effect sizes (e.g. Cohen's *d*, Pearson's *r*), indicating how they were calculated

Our web collection on statistics for biologists contains articles on many of the points above.

Software and code

Policy information about availability of computer code

Data collection	*No software was used for data collection.*
Data analysis	*All statistical analyses were performed using R Statistics (version 4.1.2) or SPSS (version 20.0). Plots were created using the ggplot2 package (version 3.3.5) R Statistics.*

For manuscripts utilizing custom algorithms or software that are central to the research but not yet described in published literature, software must be made available to editors and reviewers. We strongly encourage code deposition in a community repository (e.g. GitHub). See the Nature Portfolio guidelines for submitting code & software for further information.

Data

Policy information about availability of data

All manuscripts must include a data availability statement. This statement should provide the following information, where applicable:

- Accession codes, unique identifiers, or web links for publicly available datasets
- A description of any restrictions on data availability
- For clinical datasets or third party data, please ensure that the statement adheres to our policy

All data supporting the findings described in this manuscript are available in the article and in the Supplementary Information or/and from the corresponding author upon request. Data used to generate the main and supplementary figures are provided in the Source Data file. The patient data used in this study cannot be shared publicly due to privacy restrictions. However, in the case of noncommercial use, researchers can sign the license, complete a data access form provided at https://

github.com/RYL-gif/LensAge and contact H.L. to access the de-identified representative images. For requests from verified academic researchers, access will be evaluated by the data access committee and be granted within one month. Source data are provided with this paper.

Research involving human participants, their data, or biological material

Policy information about studies with human participants or human data. See also policy information about sex, gender (identity/presentation), and sexual orientation and race, ethnicity and racism.

Reporting on sex and gender	*The participants were recruited without sex restriction. The baseline information of sex was obtained from Chinese government-issued official resident identity card. A total of 5,127 participants were included in this study (41.2% males). We evaluated the LensAge index stratified by sex for subgroup analysis.*
Reporting on race, ethnicity, or other socially relevant groupings	*A breakdown of the participants' nationalities is reported in the list of baseline characteristics in Table 1*
Population characteristics	*All participants were aged between 20 to 96 years old. All enrolled participants were eligible for the study if they had no history of previous eye surgery, eye trauma, ocular diseases (high myopia, etc.) that can cause complicated cataracts, and long-term use of corticosteroids or other drugs that can cause drug-induced cataracts.*
Recruitment	*For diffuse-light and slit-lamp photographs, participants of 20 to 96 years old were recruited from (1) an ongoing national Chinese cataract screening program by the Chinese Medical Alliance for Artificial Intelligence (CMAAI) between April 2018 and May 2021; and (2) the retrospective hospital dataset of Zhongshan Ophthalmic Center (Sun Yat-sen University, Guangdong, China) between January 2020 and May 2021 collected from hospital admission records. For smartphone photographs, participants aged 35 to 90 years were recruited from Sun Yat-sen Memorial Hospital (Sun Yat-sen University, Guangdong, China) and community screening in Lianzhou, Guangdong, China. There are some biases: (1) The study may not included individuals with extremely poor health status across various age groups, as they were less likely to participate in. (2) Most of the participants were Chinese and it may be limited to other ethnicities and nationalities. (3) Our methods may not be appropriate for a proportion of participants with complicated cataracts or medical history of intraocular surgeries. (4) We may include participants who were unaware of their underlying and undiagnosed diseases in the relatively healthy datasets and may have influence on our models to learn the average aging characteristics among the population.*
Ethics oversight	*The study protocol was approved by the Institutional Review Board/Ethics Committee of Zhongshan Ophthalmic Center and registered on ClinicalTrials.gov (Identifier NCT05588921).*

Note that full information on the approval of the study protocol must also be provided in the manuscript.

Field-specific reporting

Please select the one below that is the best fit for your research. If you are not sure, read the appropriate sections before making your selection.

☒ Life sciences ☐ Behavioural & social sciences ☐ Ecological, evolutionary & environmental sciences

For a reference copy of the document with all sections, see nature.com/documents/nr-reporting-summary-flat.pdf

Life sciences study design

All studies must disclose on these points even when the disclosure is negative.

Sample size	*Referring to the previous studies by Long et al (doi: 10.1038/s41551-016-0024) and Wu et al. (doi: 10.1136/bjophthalmol-2019-315025), considering the requirement of samples for DL model training, and to further investigate the ability of the LensAge index to reflect aging status, an appropriate sample size was obtained in this study.*
Data exclusions	*The participants who had a history of previous eye surgery, eye trauma, ocular diseases (high myopia, etc.) that can cause complicated cataracts, or long-term use of drugs that can cause drug-induced cataracts were excluded. Those who could not undergo binocular anterior segment photographs were excluded. In addition, the images without sufficient image quality were excluded.*
Replication	*Our findings of performance of our DL models, LensAge, and the LensAge index were derived from the artificial intelligence algorithms and statistical analysis.*
Randomization	*Images for model development were randomly split into a training set (60%), a tuning set (20%), and a validation set (20%). All data were split at the individual level.*
Blinding	*Blinding was not relevant to our study because no experimental group assignment was needed in our study.*

Behavioural & social sciences study design

All studies must disclose on these points even when the disclosure is negative.

Study description	*not relevant*
Research sample	*not relevant*
Sampling strategy	*not relevant*
Data collection	*not relevant*
Timing	*not relevant*
Data exclusions	*not relevant*
Non-participation	*not relevant*
Randomization	*not relevant*

Ecological, evolutionary & environmental sciences study design

All studies must disclose on these points even when the disclosure is negative.

Study description	*not relevant*
Research sample	*not relevant*
Sampling strategy	*not relevant*
Data collection	*not relevant*
Timing and spatial scale	*not relevant*
Data exclusions	*not relevant*
Reproducibility	*not relevant*
Randomization	*not relevant*
Blinding	*not relevant*

Did the study involve field work? ☐ Yes ☒ No

Field work, collection and transport

Field conditions	*not relevant*
Location	*not relevant*
Access & import/export	*not relevant*
Disturbance	*not relevant*

Reporting for specific materials, systems and methods

We require information from authors about some types of materials, experimental systems and methods used in many studies. Here, indicate whether each material, system or method listed is relevant to your study. If you are not sure if a list item applies to your research, read the appropriate section before selecting a response.

Materials & experimental systems

n/a	Involved in the study
☒	☐ Antibodies
☒	☐ Eukaryotic cell lines
☒	☐ Palaeontology and archaeology
☒	☐ Animals and other organisms
☐	☒ Clinical data
☒	☐ Dual use research of concern
☒	☐ Plants

Methods

n/a	Involved in the study
☒	☐ ChIP-seq
☒	☐ Flow cytometry
☒	☐ MRI-based neuroimaging

Antibodies

Antibodies used: *not relevant*

Validation: *not relevant*

Eukaryotic cell lines

Policy information about cell lines and Sex and Gender in Research

Cell line source(s): *not relevant*

Authentication: *not relevant*

Mycoplasma contamination: *not relevant*

Commonly misidentified lines (See ICLAC register): *not relevant*

Palaeontology and Archaeology

Specimen provenance: *not relevant*

Specimen deposition: *not relevant*

Dating methods: *not relevant*

☐ Tick this box to confirm that the raw and calibrated dates are available in the paper or in Supplementary Information.

Ethics oversight: *not relevant*

Note that full information on the approval of the study protocol must also be provided in the manuscript.

Animals and other research organisms

Policy information about studies involving animals; ARRIVE guidelines recommended for reporting animal research, and Sex and Gender in Research

Laboratory animals: *not relevant*

Wild animals: *not relevant*

Reporting on sex: *not relevant*

Field-collected samples: *not relevant*

Ethics oversight: *not relevant*

Note that full information on the approval of the study protocol must also be provided in the manuscript.

Clinical data

Policy information about clinical studies

All manuscripts should comply with the ICMJE guidelines for publication of clinical research and a completed CONSORT checklist must be included with all submissions.

Clinical trial registration	*This study was registered on ClinicalTrials.gov (identifier: NCT05588921).*
Study protocol	*The study protocol was available at ClinicalTrials.gov (identifier: NCT05588921).*
Data collection	*For diffuse-light and slit-lamp photographs, participants aged 20 to 96 years were recruited from (1) an ongoing national Chinese cataract screening program by the Chinese Medical Alliance for Artificial Intelligence (CMAAI) between April 2018 and May 2021 with comprehensive baseline information (chronological age, sex, race, region, and occupation), anthropometric and lifestyle factors (not/formerly/currently smoking and alcohol intake status), medical history of diseases, regular physical examinations and ophthalmic examinations; and (2) the retrospective hospital dataset of Zhongshan Ophthalmic Center (Sun Yat-sen University, Guangdong, China) between January 2020 and May 2021 with comprehensive baseline information (chronological age, sex, race, region, and occupation), medical history of diseases, regular physical examinations, ophthalmic examinations, chest X-ray examinations, electrocardiographs, full blood count, and basic profile of blood collected from the hospital admission records. All enrolled participants were eligible for the study if they had no history of previous eye surgery, eye trauma, ocular diseases (high myopia, etc.) that can cause complicated cataracts, and long-term use of corticosteroids or other drugs that can cause drug-induced cataracts. The collected systemic medical histories at baseline included diabetes, hypertension, cardiovascular disease, cerebrovascular disease, cancer, and other chronic systemic diseases. All participants underwent regular physical examinations including heart rate, blood pressure, respiratory rate, height, and weight and ophthalmic examinations consisted of functional and structural examinations, including visual acuity, intraocular pressure, slit-lamp examinations, fundoscopy examinations, and cycloplegic refraction. All participants underwent binocular anterior segment photographs for diffuse-light and slit-lamp modes using a variety of slit lamps, including the BQ-900, BX-900, OVS-II, and PSL-Classic. For smartphone photographs, participants aged 35 to 90 years were recruited from Sun Yat-sen Memorial Hospital (Sun Yat-sen University, Guangdong, China) and community screening in Lianzhou, Guangdong, China. The inclusion criteria were the same as described above. Baseline information and a comprehensive medical history of diseases were also collected from questionnaires for the recruited individuals. All participants had smartphone photographs taken for both eyes with a portable slit lamp (MediWorks portable slit lamp S150, Shanghai) attached to the iPhone/Huawei smartphone. The participants in the analysis dataset were followed up from the time when the lens photographs were taken. To gather information on all-cause mortality status and date of death, questionnaires were administered by the investigators to the relatives of the participants. The duration of follow-up for each participant was calculated as the time elapsed between their baseline and the date of death or the completion of the follow-up period (July, 2023), whichever came first.*
Outcomes	*We aimed to develop DL models to generate LensAge values, which is a regression task. The MAE was predefined as the primary outcome for DL model error evaluation for regression task. For the LensAge index analysis, we aimed to evaluate the correlation between the LensAge index and the age-related events using regression models, OR, β and HR values were predefined. The results of logistic regression models were reported with adjusted ORs, and the results of linear regression models were reported with β. The results of Cox proportional hazards regression models were reported as the adjusted HR. Further, we aimed to evaluated the prediction performance of the LensAge index, and the AUC was predefined for the prediction models. The AUCs were used to evaluate the LensAge index and chronological age for predicting the occurrence of age-related diseases.*

Dual use research of concern

Policy information about dual use research of concern

Hazards

Could the accidental, deliberate or reckless misuse of agents or technologies generated in the work, or the application of information presented in the manuscript, pose a threat to:

No	Yes	
☒	☐	Public health
☒	☐	National security
☒	☐	Crops and/or livestock
☒	☐	Ecosystems
☒	☐	Any other significant area

Experiments of concern

Does the work involve any of these experiments of concern:

No	Yes	
☒	☐	Demonstrate how to render a vaccine ineffective
☒	☐	Confer resistance to therapeutically useful antibiotics or antiviral agents
☒	☐	Enhance the virulence of a pathogen or render a nonpathogen virulent
☒	☐	Increase transmissibility of a pathogen
☒	☐	Alter the host range of a pathogen
☒	☐	Enable evasion of diagnostic/detection modalities
☒	☐	Enable the weaponization of a biological agent or toxin
☒	☐	Any other potentially harmful combination of experiments and agents

Plants

Seed stocks: *not relevant*

Novel plant genotypes: *not relevant*

Authentication: *not relevant*

ChIP-seq

Data deposition

☐ Confirm that both raw and final processed data have been deposited in a public database such as GEO.

☐ Confirm that you have deposited or provided access to graph files (e.g. BED files) for the called peaks.

Data access links
May remain private before publication.: *not relevant*

Files in database submission: *not relevant*

Genome browser session (e.g. UCSC): *not relevant*

Methodology

Replicates: *not relevant*

Sequencing depth: *not relevant*

Antibodies: *not relevant*

Peak calling parameters: *not relevant*

Data quality: *not relevant*

Software: *not relevant*

Flow Cytometry

Plots

Confirm that:

☐ The axis labels state the marker and fluorochrome used (e.g. CD4-FITC).

☐ The axis scales are clearly visible. Include numbers along axes only for bottom left plot of group (a 'group' is an analysis of identical markers).

☐ All plots are contour plots with outliers or pseudocolor plots.

☐ A numerical value for number of cells or percentage (with statistics) is provided.

Methodology

Sample preparation	*not relevant*
Instrument	*not relevant*
Software	*not relevant*
Cell population abundance	*not relevant*
Gating strategy	*not relevant*

☐ Tick this box to confirm that a figure exemplifying the gating strategy is provided in the Supplementary Information.

Magnetic resonance imaging

Experimental design

Design type	*not relevant*
Design specifications	*not relevant*
Behavioral performance measures	*not relevant*

Acquisition

Imaging type(s)	*not relevant*
Field strength	*not relevant*
Sequence & imaging parameters	*not relevant*
Area of acquisition	*not relevant*

Diffusion MRI ☐ Used ☒ Not used

Preprocessing

Preprocessing software	*not relevant*
Normalization	*not relevant*
Normalization template	*not relevant*
Noise and artifact removal	*not relevant*
Volume censoring	*not relevant*

Statistical modeling & inference

Model type and settings	*not relevant*
Effect(s) tested	*not relevant*

Specify type of analysis: ☐ Whole brain ☐ ROI-based ☐ Both

Statistic type for inference (See Eklund et al. 2016)	*not relevant*
Correction	*not relevant*

Models & analysis

n/a	Involved in the study
☒	☐ Functional and/or effective connectivity
☒	☐ Graph analysis
☒	☐ Multivariate modeling or predictive analysis

Functional and/or effective connectivity: *not relevant*

Graph analysis: *not relevant*

Multivariate modeling and predictive analysis: *not relevant*
